# KCNQ Current Contributes to Inspiratory Burst Termination in the Pre-Bötzinger Complex of Neonatal Rats *in vitro*

**DOI:** 10.3389/fphys.2021.626470

**Published:** 2021-04-13

**Authors:** Ann L. Revill, Alexis Katzell, Christopher A. Del Negro, William K. Milsom, Gregory D. Funk

**Affiliations:** ^1^Neuroscience and Mental Health Institute, University of Alberta, Edmonton, AB, Canada; ^2^Department of Physiology, University of Alberta, Edmonton, AB, Canada; ^3^Women and Children’s Health Research Institute, University of Alberta, Edmonton, AB, Canada; ^4^Department of Applied Science, William & Mary, Williamsburg, VA, United States; ^5^Department of Zoology, University of British Columbia, Vancouver, BC, Canada

**Keywords:** breathing, Kv7, M-current, KCNQ, inspiratory rhythm, respiration

## Abstract

The pre-Bötzinger complex (preBötC) of the ventral medulla generates the mammalian inspiratory breathing rhythm. When isolated in explants and deprived of synaptic inhibition, the preBötC continues to generate inspiratory-related rhythm. Mechanisms underlying burst generation have been investigated for decades, but cellular and synaptic mechanisms responsible for burst termination have received less attention. KCNQ-mediated K^+^ currents contribute to burst termination in other systems, and their transcripts are expressed in preBötC neurons. Therefore, we tested the hypothesis that KCNQ channels also contribute to burst termination in the preBötC. We recorded KCNQ-like currents in preBötC inspiratory neurons in neonatal rat slices that retain respiratory rhythmicity. Blocking KCNQ channels with XE991 or linopirdine (applied *via* superfusion or locally) increased inspiratory burst duration by 2- to 3-fold. By contrast, activation of KCNQ with retigabine decreased inspiratory burst duration by ~35%. These data from reduced preparations suggest that the KCNQ current in preBötC neurons contributes to inspiratory burst termination.

## Introduction

Inspiration is the dominant, active phase of the respiratory cycle in mammals. Its rhythm emanates from the pre-Bötzinger complex (preBötC) of the ventrolateral medulla (Smith et al., 1991; Del Negro et al., 2018). The most recent models of inspiratory rhythm generation favor synaptic mechanisms where burstlets or bursts derive from the progressive recruitment and excitation of a critical subpopulation of Dbx1-derived, glutamatergic neurons and their increased synchronization in the pre-inspiratory and ultimately inspiratory phases (Del Negro et al., 2018; Ashhad and Feldman, 2020; Kallurkar et al., 2020).

What about inspiratory burst termination? Under normal physiological conditions in many vertebrates, feedback from slowly adapting pulmonary stretch receptors plays an important role in terminating inspiration (Bonham et al., 1993). Most importantly, when stretch receptor feedback is removed by vagotomy for experimental preparations or due to medical necessity in humans with heart–lung transplants, inspiration is prolonged, but it still terminates (Lumsden, 1923; Smith et al., 1990; Bonham et al., 1993). Similarly, dorsolateral pontine networks normally contribute to inspiratory burst termination, but even the prolonged inspiratory (apneustic) events that emerge when these inputs are removed self-terminate (Lumsden, 1923; Stella, 1938; von Euler et al., 1976; Foutz et al., 1988; Feldman et al., 1992; Harris and Milsom, 2003).

Synaptic inhibition between central respiratory neurons is also a significant contributor to burst termination because a block of this inhibition prolongs the inspiratory burst (Janczewski et al., 2013). Nevertheless, even when synaptic inhibition is blocked in the preBötC *in vivo* (Janczewski et al., 2013) or throughout the brainstem *in situ* (Hayashi and Lipski, 1992) or *in vitro* (Feldman and Smith, 1989; Feldman and Del Negro, 2006; Sherman et al., 2015; Cregg et al., 2017; Baertsch et al., 2018), inspiratory bursts still terminate. Synaptic inhibition influences inspiratory bursts but is not required for burst termination.

Stripped of reflex pathways and contributions attributable to synaptic inhibition, inspiratory burst termination is probably accomplished by cellular and synaptic mechanisms intrinsic to core rhythmogenic preBötC interneurons. Putative mechanisms include desynchronization amongst rhythm-generating neurons as the inspiratory burst progresses (Ashhad and Feldman, 2020), excitatory synaptic depression (Guerrier et al., 2015; Kottick and Del Negro, 2015), and outward currents that activate due to inspiratory burst activity and thus hasten burst termination and promote transient post-burst refractoriness.

Ca^2+^-activated K^+^ current (I_K(Ca)_) contributes to burst termination in many vertebrate central pattern generator (CPG) networks (Grillner, 2006). However, I_K(Ca)_ does not appear to contribute to burst termination in the preBötC because its block has only minor and variable effects on network rhythm and burst pattern (Onimaru et al., 2003; Crowder et al., 2007; reviewed in Rajani et al., 2016).

There is evidence for three cellular mechanisms that contribute to burst termination, including Na^+^-dependent K^+^ current (I_K-Na_), Na^+^/K^+^ ATPase pump current, and ATP-dependent K^+^ current (I_K-ATP_; Del Negro et al., 2009; Krey et al., 2010). Whole-cell current-clamp recordings show that these currents contribute to the post-inspiratory burst hyperpolarization at the level of individual neurons, but effects at the network level are more difficult to interpret. The predicted effect of blocking mechanisms involved in burst termination is an increase in burst duration, but antagonists of these ion channels decrease burst duration (Krey et al., 2010), possibly due to additional effects on resting membrane potential.

Outward current attributable to KCNQ channels (Selyanko et al., 1992; Delmas and Brown, 2005; Honigsperger et al., 2015; Greene et al., 2017), initially dubbed M-current (Brown and Adams, 1980), may also contribute to burst termination in the preBötC. There are five KCNQ genes, *KCNQ1–5*, but transcripts for *KCNQ2/3*, which encode for the subunits Kv7.2 and Kv7.3 that feature most prominently in establishing the KCNQ current (Wang et al., 1998), are expressed by preBötC neurons (Hayes et al., 2017; Wei and Ramirez, 2019). Pharmacological potentiation of KCNQ currents slows inspiratory-related frequency *in vitro* and breathing frequency *in vivo* (Wei and Ramirez, 2019). In contrast, inhibition of KCNQ channels does not affect baseline inspiratory-related frequency in mice *in vitro* or breathing frequency *in vivo*. Inhibition of preBötC KCNQ channels does, however, partially counteract the well-known opioid-mediated depression in breathing through presynaptic mechanisms, even though these channels are not directly modulated by opioid receptor signaling (Wei and Ramirez, 2019). Human patients with mutations in KCNQ2/3 genes often develop neurological disorders associated with apnea (Ronen et al., 1993; Kato et al., 2013; Grinton et al., 2015). Finally, homozygous KCNQ2 knockout mice typically die in the first day of life due to respiratory insufficiency that may have a central etiology (Watanabe et al., 2001).

Therefore, we isolated the preBötC core oscillator in rhythmically active slices with the goal of evaluating whether KCNQ-mediated outward current may play a role in inspiratory burst termination.

## Materials and Methods

### Animals and Preparations

Male and female Sprague-Dawley neonatal rat pups ranging in age from postnatal day (P) 1 to 4 were obtained from timed-pregnant dams from Charles River or from Laboratories and Science Animal Support Services (University of Alberta). Animals were housed in University of Alberta’s Health Sciences Laboratory Animal Services and maintained on a 12-h light/dark schedule with *ad libitum* access to food and water.

Rhythmic medullary slice preparations were prepared as described previously (Smith et al., 1991; Ruangkittisakul et al., 2006; Lorier et al., 2007). Briefly, rat pups were anesthetized *via* inhalation of isoflurane. Once surgical plane anesthesia was verified by lack of withdrawal reflexes, animals were immediately decerebrated. The neuraxis was isolated in a dissection chamber containing cold (5–10°C) artificial cerebrospinal fluid (aCSF) composed of the following (in mM): 120 NaCl, 3 KCl, 1.25 NaH_2_PO_4_, 1 CaCl_2_, 2 MgSO_4_, 26 NaHCO_3_, and 20 d-glucose, equilibrated with 95% O_2_–5% CO_2_. The neuraxis was pinned to a wax chuck or glued to an agar block that was placed in the vice of a vibratome (Leica VT-1000S, or Leica VT-1200S, Concord, ON, Canada). Serial sections (100–300 μm) were then cut in the rostral to caudal direction and trans-illuminated to visualize anatomical landmarks identifying the rostral border of the preBötC. When the compact division of the nucleus ambiguus was no longer evident and the rostral margin of the inferior olive appeared in the thin section, we then cut a single 700-μm slice. The rostral border of the slice was ~0.35 mm caudal to the caudal aspect of the facial nucleus, which placed the caudal edge of the slice just caudal to the obex (Ruangkittisakul et al., 2006). The rhythmic slice contained the preBötC, rostral ventral respiratory group (rVRG), most of the XII motor nuclei, and the rostral XII nerve rootlets. For experiments that examined the effects of drugs on inspiratory network activity recorded from the XII nerves, slices were pinned rostral surface up in a Sylgard-coated recording chamber (volume, 10 ml) and perfused with aCSF that was recirculated at a flow rate of 9.5 ml/min. For whole-cell recording experiments, rhythmic slices were placed rostral surface up in the recording chamber (volume, 1 ml) of an upright microscope (Zeiss Axioskop 2 FS Plus, Toronto, ON, Canada) that was equipped with infrared-enhanced differential interference contrast optics (IRDIC). Slices were held in place by a U-shaped platinum frame with a mesh of parallel nylon fibers, i.e., a “harp,” and perfused with aCSF at a flow rate of 1–2 ml/min.

We elevated extracellular K^+^ concentration ([K^+^]_o_) to 9 mM because spontaneous rhythmicity at basal [K^+^]_o_ of 3 mM decays after ~2 h, whereas slices with elevated [K^+^]_o_ retain rhythmicity for ~5 h, allowing for drug protocols that require prolonged washouts exceeding 2 h (Ruangkittisakul et al., 2006; Funk and Greer, 2013).

### Electrophysiological Recording Methods

#### XII Nerve Recording

Inspiratory-related output of the rhythmic slice was recorded from a single hypoglossal (XII) nerve rootlet using a glass suction electrode (70–90 μm I.D.). XII nerve signals were amplified (10,000×), band-pass filtered (300 Hz to 1 kHz; A-M Systems, Carlsborg, WA, United States), full-wave rectified, integrated (*τ* = 50 ms, Moving Averager, CWE Inc., Ardmore, PA, United States), and displayed on a computer monitor using AxoScope 9.2 software (pCLAMP Suite, Molecular Devices, Sunnyvale, CA, United States). Data were saved using a Digidata 1322 A/D board (Molecular Devices) and analyzed off-line using AxoScope and Clampfit software.

#### Whole-Cell Recording

Whole-cell recordings were made from inspiratory-modulated preBötC neurons (Rekling et al., 1996). A horizontal puller (P-97, Sutter Instruments, Novato, CA, United States) was used to pull whole-cell recording pipettes (3–4.5 MΩ) from filamented borosilicate glass (Clark/WPI, 1.2 mm O.D.). Patch pipettes were filled with intracellular solution containing (in mM) 140 K^+^-gluconate, 5 NaCl, 0.1 EGTA, 10 HEPES, 1 MgCl_2_, 1 glucose, and 1 Mg_2_ATP. Values of membrane potential (V_m_) have not been corrected for the liquid junction potential of −14.1 mV (calculated using Clampex, pCLAMP Suite) that is associated with these solutions. Osmolarity was adjusted to 290–300 mOsm with sucrose, and pH was adjusted to 7.2–7.3 using KOH. Whole-cell signals (membrane current or voltage) were amplified and filtered with a patch-clamp amplifier (2–5 kHz low-pass filter, MultiClamp 700A, Axon Instruments) and sampled at 10 kHz *via* an analog-to-digital converter (Digidata 1322 A/D, Molecular Devices). Series resistance (R_s_) was estimated and tracked throughout the experiment under voltage-clamp conditions. R_s_ measured 14.0 ± 1.4 MΩ (range: 12–16 MΩ). We excluded recordings in which R_s_ varied by more than 20%.

### Drugs and Drug Application

Drugs were prepared as concentrated stock solutions in distilled water or dimethyl sulfoxide (DMSO) and diluted with aCSF containing 9 mM K^+^ to the final concentration. Drugs included the following:

- [Sar^9^-Met(O_2_)^11^]-substance P (SP), which is a neurokinin-1 receptor (NK1R) agonist, was microinjected at 1 μM (Tocris, Bristol, UK).
- 10,10-*bis*(4-pyridinyl-methyl)-9(10H)-anthracenone (XE991), which is a KCNQ channel blocker (Brown and Passmore, 2009). XE991 was bath applied at 0.1–1.0 μM and locally applied at 10 μM for networks studies and 100 μM for whole-cell experiments. It was prepared from stock dissolved in DMSO (Sigma-Aldrich, St. Louis, MO, United States).
- 1,3-Dihydro-1-phenyl-3,3-*bis*(4-pyridinylmethyl)-2*H*-indol-2-one dihydrochloride (linopirdine), a KCNQ channel blocker (Brown and Passmore, 2009), was bath applied at 3 μM (Tocris).
- Ethyl[2-amino-4-[[(4-fluorophenyl)methyl]amino]phenyl]carbamate (retigabine), a KCNQ channel activator, was bath applied at 2–3 μM from stock dissolved in DMSO (Alomone Labs, Jerusalem, Israel).
- Octahydro-12-(hydroxymethyl)-2-imino-5,9:7,10a-dimethano-10aH-[1,3]dioxocino[6,5-d]pyrimidine-4,7,10,11,12-pentol [tetrodotoxin (TTX), citrate salt] was bath applied at 0.5 μM (Alomone Labs).
- 4-Ethylphenylamino-1,2-dimethyl-6-methylaminopyrimidinium chloride (ZD7288), which blocks the hyperpolarization-activated mixed cation current (I_h_), was bath applied at 10 μM (Tocris).

The final concentration of DMSO in the aCSF or in the injection solutions never exceeded 0.003% for bath application of retigabine or XE991 and 0.02% for local application of XE991.

Drugs were either bath-applied or locally microinjected into the preBötC *via* pressure ejection from triple-barreled glass micropipettes (4–6 μm O.D. per barrel) pulled from borosilicate glass capillaries (cat no. 3B120F-4, WPI, Sarasota, FL, United States) using a vertical puller (P-35, Sutter Instruments). Drug concentration in the ejection pipette solution must be ∼10-fold greater than the bath-applied concentration to produce similar effects (Liu et al., 1990). For studies in which drugs were injected directly into the preBötC, the preBötC was first functionally identified by mapping the response to local injections of SP. In the site at which SP (1 μM, 10 s) evoked a rapid (next cycle), transient increase in inspiratory frequency that was at least double baseline was considered the preBötC core (see Zwicker et al., 2011).

For whole-cell recording experiments, drug ejection pipettes were placed 25–50 μm upstream of the target neuron at the superficial slice surface. With this arrangement, recorded neurons were typically 30–70 μm below the slice surface and 50–120 μm from the injection pipette tip. This placement, along with maintaining ejection pressure below 5 psi, was important to limit mechanical artifacts. In addition, membrane current was monitored carefully at pressure onset for abrupt, non-recoverable steps in holding current synchronous with pressure onset indicative of mechanical artifacts. Data were rejected if mechanical artifacts were noticed or R_s_ changed by more than 20%. We have previously established that vehicle solutions containing DMSO at concentrations 0.2% (10-fold higher than used here) are without noticeable effect on membrane properties or inspiratory synaptic drive currents/potentials/firing behavior (Funk et al., 1995; Ireland et al., 2012).

Note that after each experiment in which XE991 was bath-applied *via* the perfusion system, the inflow and outflow tubing (Tygon) was rinsed with at least 500 ml of ethanol followed by 2 L of double-distilled deionized water; and glass reservoirs and the recording chamber were thoroughly washed with ethanol and rinsed with double-distilled deionized water to remove residual XE991. Without this procedure, the baseline burst duration produced by the rhythmic slices on subsequent days would gradually increase during the first hour of slice recording (i.e., prior to the start of the experiment) to levels greater than twice the normal baseline values (i.e., >2,000 ms compared with a normal burst duration <1,000 ms). Thus, every experiment began with a 1-h recording of baseline activity to ensure that there were no residual effects of XE991. If a residual effect was observed, then we discarded those data, cleaned the recording chamber, and replaced the tubing.

### Data Analysis and Statistical Comparisons

All measurements relating to frequency (inspiratory burst duration and inspiratory frequency) are reported as absolute values and relative to control, with mean ± standard deviation. Inspiratory XII burst amplitude is reported relative to control. The units of burst amplitude recorded from a suction electrode are not reported because they have no physiological meaning except as a relative comparison before/after drug application, such as in this context. Statistical tests were performed on raw data except for burst amplitude where relative values were compared. Student’s paired t test was used to compare parameters between two groups. One-way analysis of variance (ANOVA) with repeated measures (RM; used when no data were missing from any measurement category, i.e., when XE991 was bath-applied), or a mixed-effects model (used when data were missing from a measurement category, i.e., when linopirdine or retigabine were bath-applied) followed by Tukey’s multiple comparison *post hoc* test (Prism 4.2 or 9.0.1, GraphPad Software, San Diego, CA, United States), was used to compare parameters between three or more groups. One-way ANOVA followed by Tukey’s multiple comparison *post hoc* test was used to compare parameters between [K^+^]_e_ elevation, XE991, and retigabine. Differences between means were considered significant if *p* < 0.05.

## Results

**Figure 1:**
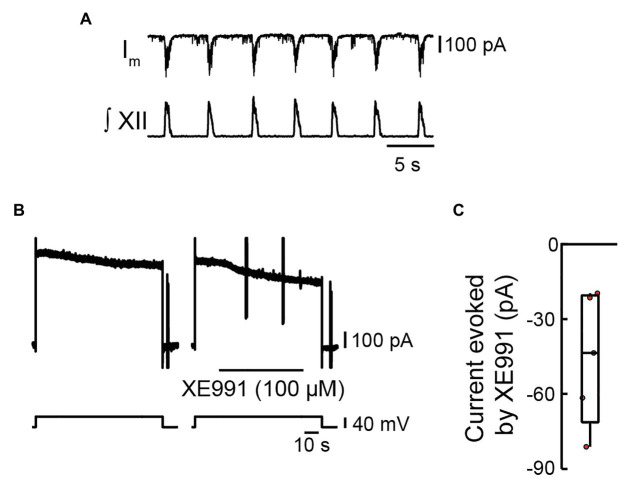
Effects of locally applied XE991 on membrane current in inspiratory pre-Bötzinger complex (preBötC) neurons. **(A)** Voltage-clamp recording from an inspiratory preBötC neuron showing rhythmic synaptic inputs in phase with bursts of inspiratory motor output recorded from the XII nerve prior to bath application of tetrodotoxin (TTX; 0.5 μM) and ZD7288 (10 μM). **(B)** Voltage-clamp recording from the same neuron in **(A)** after the network was silenced *via* bath application of TTX and ZD7288, showing the current responses evoked by a depolarizing step from −60 to −20 mV for 90 s during control (left panel) and when XE991 is locally applied 20 s into the 90-s depolarizing step (right panel). **(C)** Group data showing current evoked in inspiratory preBötC neurons by local application of XE991 during a depolarizing step to −20 mV (box plot: median and interquartile range; whiskers: 10th and 90th percentiles) and individual values (red circles, *n* = 5).

We obtained voltage-clamp recordings from preBötC inspiratory neurons (input resistance measured 213 ± 144 MΩ, *n* = 5), as indicated by the presence of inward synaptic currents coincident with bursts of inspiratory motor output on the XII nerve (Figure 1A). We bath-applied TTX (0.5 μM) to block sodium currents and ZD7288 (10 μM) to block the hyperpolarization-activated mixed cation current (I_h_). The activation of sodium currents or deactivation of I_h_ (Thoby-Brisson et al., 2000) would otherwise obscure KCNQ current, which we aimed to evoke with our voltage-clamp protocol. We stepped the membrane from −60 to −20 mV for 90 s to evoke KCNQ current, which does not inactivate (Figure 1B). Peak current measured ~600 pA, which then decreased at a constant rate of ~1 pA/s over 90 s. We assume the outward currents that declined at ~1 pA/s reflected non-KCNQ outward currents. XE991 blocks KCNQ in the open configuration (Greene et al., 2017), so we could only employ this drug after having opened the channel. The subsequent step evoked outward current matching the magnitude of the outward current at the termination of the first step. We monitored that current for 14–20 s, which again decreased at the rate of ~1 pA/s. We then applied XE991 (100 μM) locally (70–76 s, i.e., 90 s – the duration of monitoring). XE991 evoked an inward current (by blocking open KCNQ channels) that stabilized to the initial rate of decay. The current that developed between the onset of the XE991 application and the end of the −20-mV pulse was subtracted from the current that developed over the same time period in the control pulse to calculate the XE991-sensitive current.

XE991 reduced the outward current in five of five inspiratory neurons by −45 ± 26 pA (Figure 1C). With the use of Ohm’s law, this change in current was calculated to result in a −6.8 ± 1.5-mV hyperpolarization of membrane potential.

**Figure 2:**
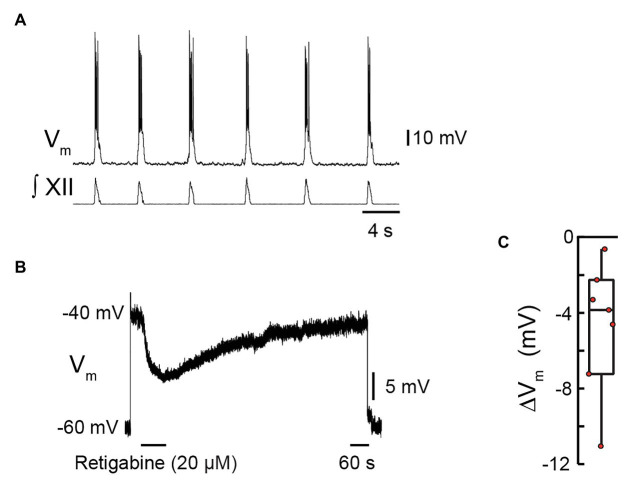
Effects of retigabine, a KCNQ channel activator, on membrane potential of inspiratory pre-Bötzinger complex (preBötC) neurons. Current clamp recording from a preBötC inspiratory neuron showing depolarization and bursts of action potentials in phase with inspiratory bursts recorded from the XII nerve prior to bath application of tetrodotoxin (TTX; 0.5 μM) and ZD7288 (10 μM). **(B)** Current-clamp recording from the same neuron in **(A)** in the presence of TTX and ZD7288, showing that when the neuron is depolarized from −60 mV (holding) to −40 mV *via* injection of D.C. current, local application of retigabine (20 μM, 80 s) caused membrane hyperpolarization. **(C)** Group data showing maximum change in membrane potential evoked by local application of retigabine (box plot: median and interquartile range; whiskers: 10th and 90th percentiles) and individual values (red circles), *n* = 7.

If KCNQ contributes to inspiratory burst termination, then its activation should hyperpolarize preBötC inspiratory neurons from potentials achieved at the end of an inspiratory cycle. We tested this idea in current-clamp recordings from seven preBötC inspiratory neurons synaptically isolated by 0.5 μM of TTX in the presence of 10 μM of ZD7288 (Figure 2A). Retigabine modulates KCNQ allosterically by hyperpolarizing its voltage dependence of activation, which accelerates, prolongs, and increases KCNQ channel openings in response to depolarization (Gunthorpe et al., 2012). The preBötC neurons were depolarized to −40 mV to approximate the membrane potential attained during the inspiratory drive potential. Retigabine (20 μM) was applied locally for 90 s. Figure 2B shows that retigabine elicited a hyperpolarization of −11.1 mV that recovered back to the initial holding potential several minutes after terminating the injection. Locally applied retigabine hyperpolarized preBötC inspiratory neurons by −4.7 ± 3.5 mV (Figure 2C, *n* = 7, *p* = 0.01).

Note that the response to retigabine does not reveal the maximum KCNQ-mediated hyperpolarization but rather hyperpolarization achieved by the allosteric potentiation of the KCNQ current. Nevertheless, a response to retigabine does confirm the presence of KCNQ channels in preBötC inspiratory neurons and their influence on membrane potential in the range of −60 to −40 mV.

We next examined the impact of modulating KCNQ channels on inspiratory network activity. If KCNQ contributes to inspiratory burst termination, then KCNQ inhibition should increase burst duration, whereas its potentiation should reduce burst duration.

**Figure 3:**
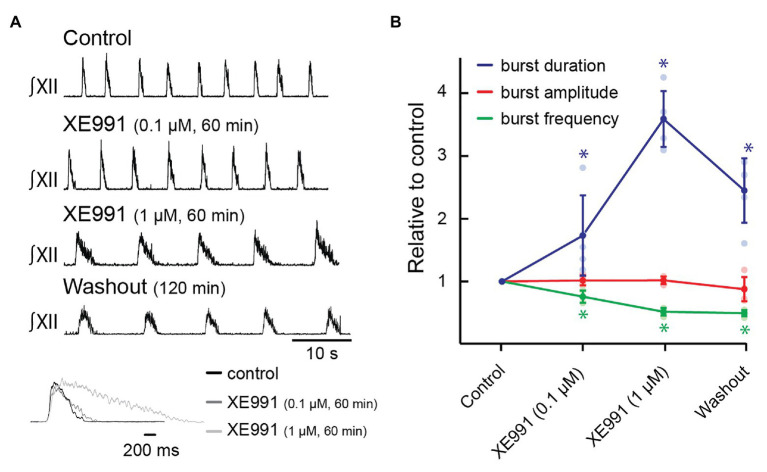
Effects of blocking KCNQ channels with XE991 on inspiratory burst parameters in the rhythmic slice preparation. **(A)** Rectified, integrated recordings from XII nerve rootlets demonstrating inspiratory burst parameter changes in the presence of bath application of XE991. Inset shows an average of 10 inspiratory bursts in control (black), after 60 min of bath application of XE991 at 0.1 μM (dark gray), and after 60 min of bath application of XE991 at 1 μM (light gray). **(B)** Group data from XII nerve rootlet recordings where XE991 was applied to the bath at 0.1 and then 1 μM, *n* = 5. ^*^p < 0.05, repeated measures (RM) ANOVA with Tukey’s multiple comparisons *post hoc* test.

First, we tested whether inhibiting KCNQ increased burst duration. Rhythmic slices were exposed to XE991 (0.1 and 1.0 μM) *via* bath superfusion. Preliminary data indicated that XE991 required >30 min to achieve steady-state effects on network rhythm, so we assessed inspiratory activity after 60 min of incubation at each concentration. Figure 3A illustrates that XII motor output burst duration increased and that frequency decreased at both concentrations, although the effects on burst duration were stronger. The inset shows the burst profile of XII inspiratory-related output averaged from 10 consecutive cycles in control, 0.1, and 1 μM of XE991.

The profile and duration of the XII motor output are reliable indicators of collective activity in the preBötC (Del Negro et al., 2001), although the absence of XII motor output does not indicate lack of collective activity in the preBötC (Kam et al., 2013; Ashhad and Feldman, 2020; Kallurkar et al., 2020).

Group data indicate that XE991 applied at 0.1 and 1.0 μM significantly increased inspiratory burst duration from 1,001 ± 169 ms at baseline to 1,680 ± 459 ms (173 ± 64% of control, Figure 3B, *n* = 5, *p* = 0.03), and 3,541 ± 307 ms (359 ± 44% of control, Figure 3B, *n* = 5, *p* < 0.0001), respectively. Inspiratory burst frequency decreased from 11.9 ± 1.7 to 9.1 ± 1.9 bursts/min to 76 ± 10% of control (Figure 3B, *n* = 5, *p* = 0.002) in 0.1 μM of XE991 and to 6.1 ± 0.3 bursts/min in 1.0 μM of XE991 (51 ± 6% of control, Figure 3B, *n* = 5, *p* < 0.0001). XII inspiratory burst amplitude was unaffected by bath application of XE991 at 0.1 μM (101 ± 8% of baseline amplitude, Figure 3B, *n* = 5, *p* = 0.98) or at 1 μM (102 ± 6% of baseline amplitude, Figure 3B, *n* = 5, *p* = 0.99).

The effects of XE991 on inspiratory rhythm were partially reversible. After 120 min of washout, burst duration returned toward control values (2,387 ± 117 ms, 245 ± 23% of control, Figure 3B, *n* = 5, *p* = 0.0001), but burst frequency did not recover (5.9 ± 0.4 bursts/min, 49 ± 2% of control, Figure 3B, *n* = 5, *p* < 0.0001). Slow and incomplete recovery from XE991 and related compounds has been reported previously (Martire et al., 2004; Yue and Yaari, 2004; Gu et al., 2005; Peters et al., 2005; Yeung and Greenwood, 2005; Wladyka and Kunze, 2006). Incomplete recovery likely reflects long-term binding, and recovery of a KCNQ current after removal of XE991 in other systems requires insertion of new KCNQ channels into the membrane (Greene et al., 2017).

**Figure 4:**
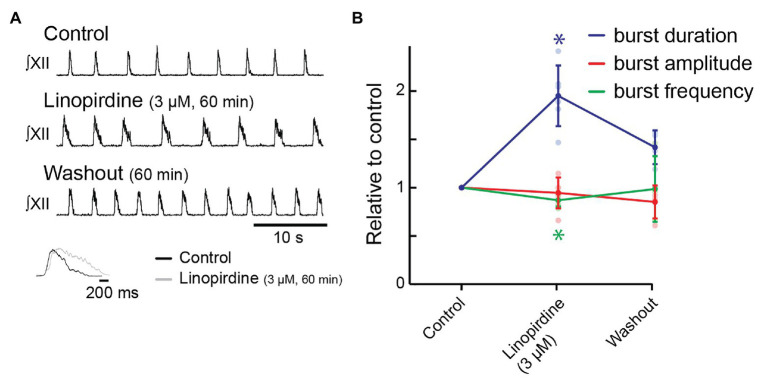
Effects of blocking KCNQ channels with linopirdine on inspiratory burst parameters in the rhythmic slice preparation. **(A)** Rectified, integrated recordings from XII nerve rootlets demonstrating inspiratory burst parameter changes in the presence of bath application of linopirdine. Inset shows an average of 10 inspiratory bursts in control (black) and after 60 min of bath application of linopirdine (light gray). **(B)** Group data from XII nerve rootlet recordings where linopirdine was applied to the bath at 3 μM, *n* = 6, *n* = 4 washout. ^*^p < 0.05, mixed-effects model with Tukey’s multiple comparisons *post hoc* test.

We then tested the effects on inspiratory behavior of linopirdine (3 μM), another open blocker of KCNQ channels whose affinities for the five KCNQ subunits differ from those of XE991 (Yue and Yaari, 2004; Greene et al., 2017). Linopirdine also required >30 min to develop steady-state effects. Bath-applied linopirdine (60 min) significantly increased burst duration from 667 ± 124 to 1,284 ± 221 ms (195 ± 31% increase, Figures 4A,B, *n* = 6, *p* = 0.001). Frequency decreased from 13.3 ± 1.3 to 11.6 ± 1.6 bursts/min (87 ± 7% of control, Figures 4A,B, *n* = 6, *p* = 0.009) in linopirdine. Linopirdine did not affect inspiratory burst amplitude (95 ± 16% of control amplitude after 60 min, Figure 4B, *n* = 6, *p* = 0.28). The inset shows the burst profile of XII inspiratory-related output averaged from 10 consecutive cycles in control and 3 μM of linopirdine.

We achieved a substantial washout in four preparations. Inspiratory burst duration recovered to 960 ± 220 ms after 60 min (141 ± 18% of control, Figure 4B, *n* = 4, *p* = 0.02). Frequency recovered completely (12.9 ± 4.3 bursts/min; 99 ± 3% of control, Figure 4B, *n* = 4, *p* = 0.97).

**Figure 5:**
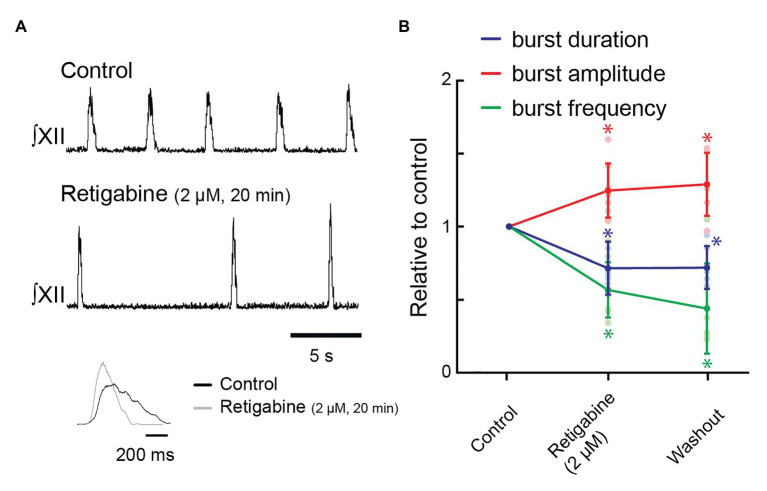
Effects of activating KCNQ channels with retigabine in the bath on inspiratory burst parameters in the rhythmic slice preparation. **(A)** Rectified, integrated recordings from XII nerve rootlets demonstrating inspiratory burst parameter changes in the presence of bath application of retigabine. Inset shows an average of 10 inspiratory bursts in control (black) and after 60 min of bath application of 2 μM of retigabine (gray). **(B)** Group data from XII nerve rootlet recordings where retigabine was applied to the bath at 2 μM, *n* = 8 for control and 2 μM of retigabine, *n* = 6 washout. ^*^p < 0.05, mixed-effects model with Tukey’s multiple comparisons *post hoc* test.

Next, we tested whether potentiation of KCNQ would decrease burst duration using bath-applied retigabine. Retigabine (2 μM, 20 min) reduced inspiratory burst duration from 560 ± 76 to 396 ± 83 ms (72 ± 18% of control, Figures 5A,B, *n* = 8, *p* = 0.007); decreased burst frequency from 13.1 ± 3.3 to 7.4 ± 2.7 bursts/min (57 ± 19% of control frequency, Figures 5A,B, *n* = 8, *p* = 0.002); and increased burst amplitude to 125 ± 19% of control (Figures 5A,B, *n* = 8, *p* = 0.03).

Rhythmic inspiratory activity ceased in four of eight preparations when retigabine was increased to 3 μM. In the remaining four preparations, inspiratory burst frequency further slowed and inspiratory burst duration further decreased in three of these preparations.

After 60 min of washout, rhythmic inspiratory activity returned in two of four preparations in which inspiratory rhythm had stopped, while burst frequency increased in the remaining four preparations. However, none of the inspiratory burst parameters recovered fully: inspiratory burst duration was 404 ± 29 ms (72 ± 6% of control, Figure 5B, *n* = 6, *p* = 0.01), burst frequency was 5.2 ± 1.3 bursts/min (44 ± 13% of control, Figure 5B, *n* = 6, *p* = 0.02), and burst amplitude was 129 ± 9% of control (Figure 5B, *n* = 6, *p* = 0.03).

Bath-applied drugs may impact microcircuits throughout the slice. Therefore, changes in XII burst duration could reflect direct actions on preBötC rhythmogenic interneurons (Del Negro et al., 2018), output-related preBötC interneurons (Kam et al., 2013; Cui et al., 2016; Ashhad and Feldman, 2020; Kallurkar et al., 2020), neuronal populations upstream from the preBötC network that provide modulatory inputs (Ptak et al., 2009), or network elements downstream of the preBötC, specifically XII premotoneurons (Koizumi et al., 2008; Revill et al., 2015) or XII motoneurons. Therefore, to ascertain whether KCNQ modulation and its effects on XII motor output were mediated (at least in part) by preBötC neurons, we locally injected XE991 in the preBötC unilaterally and assessed its effects on inspiratory rhythm and pattern.

**Figure 6:**
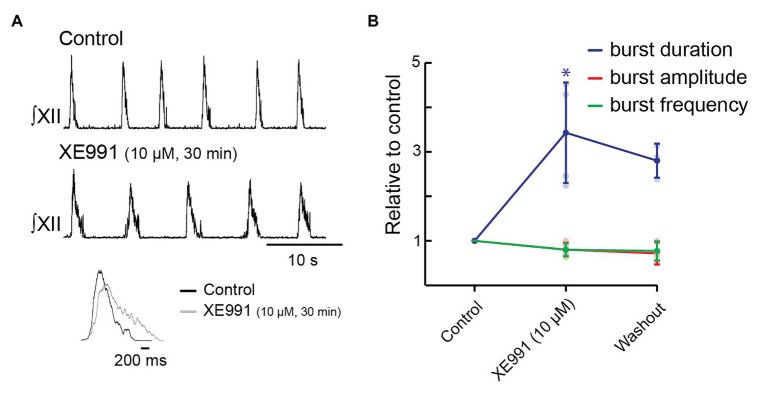
Effects of blocking KCNQ channels with XE991 locally within pre-Bötzinger complex (preBötC) on inspiratory burst parameters in the rhythmic slice preparation. **(A)** Rectified, integrated recordings from XII nerve rootlets demonstrating inspiratory burst parameter changes in the presence of local application of XE991 as a series of 5-s pulses for 30 min. **(B)** Group data from XII nerve rootlet recordings when XE991 was applied locally within preBötC, *n* = 6 for control and 10 μM of XE991 after 30 min, *n* = 4 washout. ^*^p < 0.05, paired *t*-test.

Because the effects of bath-applied XE991 took at least 30 min to appear, we locally injected XE991 (10 μM) for 30 min with a 5-s on/off duty cycle, as this method of prolonged vehicle injection *in vitro* does not disrupt inspiratory rhythm (Rajani et al., 2018). Inspiratory burst duration increased from 768 ± 189 to 2,543 ± 662 ms (343 ± 113% increase, Figures 6A,B, *n* = 5, *p* = 0.001). We achieved partial washout in four preparations; inspiratory burst duration recovered 15% to 2,165 ± 825 ms (Figure 6B, *n* = 4, *p* = 0.0012). Neither inspiratory burst frequency (13.1 ± 2.0 bursts/min in control; 10.4 ± 2.2 bursts/min in XE991, Figure 6B, *n* = 5, *p* = 0.14, and 10.6 ± 2.3 after washout, Figure 6B, *n* = 4, *p* = 0.18) nor XII burst amplitude (80 ± 15% of control after 30 min in XE991, Figure 6B, *n* = 5, *p* = 0.16, and 77 ± 22% of control after washout, Figure 6B, *n* = 4, *p* = 0.068) was significantly affected by local injection of XE991 into the preBötC. The inset shows the burst profile of XII inspiratory-related output averaged from 10 consecutive cycles in control and 10 μM of XE991.

KCNQ activates below −60 mV and reaches full activation near zero mV (Marrion, 1997; Brown and Passmore, 2009; Gunthorpe et al., 2012). Thus, in these experiments where slices are perfused with aCSF containing extracellular K^+^ ([K^+^]_e_) at 9 mM and baseline membrane potential of preBötC neurons is in the range of −50 mV (see experiments associated with Figures 1, 2), KCNQ will be partially activated under baseline conditions and become further activated with depolarization during the inspiratory burst. Thus, in addition to affecting the dynamics of the network by altering burst profile during the inspiratory phase, KCNQ will contribute to resting membrane potential. It is therefore challenging to determine whether the effects of KCNQ modulation on inspiratory output described above are due to its effect on resting membrane potential or the time- and voltage-dependent properties of KCNQ that will determine its pattern of activation during an inspiratory burst. To try and separate these effects, we compared the effects on burst duration and frequency that were produced by changing membrane potential through alterations in [K^+^]_e_ from 6 to 9 mM with those produced by inhibiting or activating KCNQ with XE991 (and linopirdine) or retigabine, respectively. This change in [K^+^]_e_ was selected because it produced a change in the membrane potential of preBötC neurons (~6-mV depolarization) similar in absolute value to that produced by retigabine (~5-mV hyperpolarization) or XE991 (~7-mV depolarization).

Elevating [K^+^]_e_ from 6 to 9 mM depolarized preBötC neurons and XII motoneurons on average from −57 ± 10.8 to −51 ± 3.8 mV (preBötC, *n* = 5), and −53 ± 5.1 to −47 ± 2.8 (XII, *n* = 9), an average of ~6 mV. If the effects of KCNQ modulators on inspiratory bursts are solely due to their effects on membrane potential, then the depolarizing actions of XE991 should match those evoked by the change in [K^+^]_e_, while the effects of retigabine should be the opposite.

**Figure 7:**
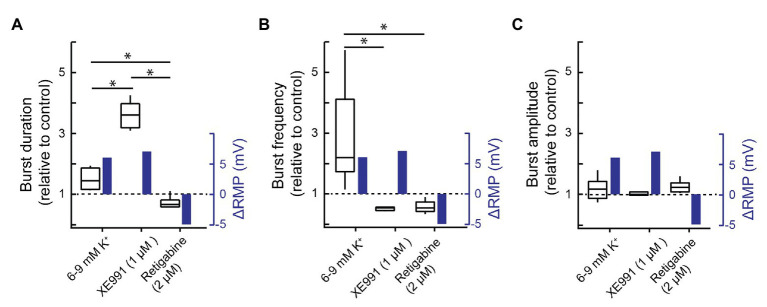
Effects of altering membrane potential vs. KCNQ modulation on inspiratory burst behavior. **(A)** Group data showing the relative change in XII inspiratory burst duration (left-hand *y*-axis) and the change in resting membrane potential (blue, right-hand *y*-axis) evoked by increasing [K^+^]_e_ from 6 to 9 mM (*n* = 4) or bath applying XE991 (1 μM, *n* = 5) or retigabine (2 μM, *n* = 8). **(B)** Group data showing the effects of increasing [K^+^]_e_ (6–9 mM, *n* = 11) or bath applying XE991 (1 μM, *n* = 5) or retigabine (2 μM, *n* = 8) on inspiratory burst frequency (relative to control) and resting membrane potential. **(C)** Group data showing effects of increasing [K^+^]_e_ (6–9 mM, *n* = 13) or bath applying XE991 (1 μM, *n* = 5) or retigabine (2 μM, *n* = 8) on inspiratory burst amplitude and resting membrane potential. The change in resting membrane potential evoked by increasing extracellular K^+^ concentration from 6 to 9 mM was measured in pre-Bötzinger complex (preBötC) inspiratory neurons (*n* = 5); the change in resting membrane potential associated with XE991 was estimated using Ohm’s law and the XE991-evoked current measured under voltage clamp and the input resistance of the recorded neurons (*n* = 5, Figure 1); the retigabine-evoked change in resting membrane potential was measured at −40 mV (Figure 2, *n* = 7). ^*^p < 0.05, one-way ANOVA with Tukey’s multiple comparisons *post hoc* test.

Figure 7 shows the effects of elevated [K^+^]_e_ on resting membrane potential, burst duration, burst frequency, and burst amplitude next to the effects of XE991 and retigabine on these same measurements. [K^+^]_e_ elevation and XE991 have similar effects on baseline membrane potential yet disparate effects on burst duration and frequency. Elevating [K^+^]_e_ increased burst duration significantly by ~40% (from 577 ± 187 to 810 ± 80 ms, Figure 7A, *n* = 4, *p* = 0.04), but XE991 caused a >300% increase in burst duration, significantly greater than K^+^-mediated depolarization (Figure 7A, three groups, 22 degrees of freedom, *p* < 0.0001).

In addition, while elevating [K^+^]_e_ from 6 to 9 mM increased burst frequency more than 2-fold from 6.6 ± 3.1 to 14.6 ± 2.0 bursts/min (Figure 7B, *n* = 11, *p* = 1.6E−5), XE991 and retigabine both decreased frequency from 11.9 ± 1.7 to 9.1 ± 1.9 (*n* = 5) and 13.1 ± 3.3 to 7.4 ± 2.7 (*n* = 8) bursts/min (Figure 7B, three groups, 22 degrees of freedom, *p* = 0.002 and *p* = 0.0006, respectively). Burst amplitude was insensitive to drug treatments (Figure 7C, three groups, 22 degrees of freedom, *p* = 0.37). Thus, changes in XII output produced by KCNQ manipulations cannot be attributed solely to changes in baseline membrane potential.

## Discussion

Synaptic depression (Rubin et al., 2009; Guerrier et al., 2015; Kottick and Del Negro, 2015) and activity-dependent, Na^+^-linked mechanisms including Na^+^/K^+^ ATPase pumps, I_K-Na_, and I_KATP_ (Del Negro et al., 2009; Krey et al., 2010) contribute to burst termination. Counterintuitively, pharmacological inhibition of those currents reduces, rather than lengthens, inspiratory burst duration. So, clearly, there are additional contributors to burst termination. We focused on KCNQ current because preBötC neurons express transcripts for KCNQ channels, it shapes burst profile in other networks, its voltage dependence and kinetics are suitable for burst termination, and Ca^2+^-activated K^+^ currents have been excluded as contributors to inspiratory burst termination in the preBötC (reviewed in Rajani et al., 2016).

Here, our measurements of XII nerve burst duration *in vitro* show that blocking KCNQ (throughout the slice or locally within the preBötC) prolongs bursts, whereas activating KCNQ (throughout the slice) shortens burst duration and stops rhythmicity. We propose that KCNQ current represents a heretofore missing contributor to inspiratory burst termination.

### Mechanisms Through Which KCNQ Modulation Influences Inspiratory Activity

Slow (tens of milliseconds) KCNQ activation is suitable for recruitment during inspiratory bursts, lasting ~300 ms, contributing to spike frequency adaption, and causing progressive membrane hyperpolarization (Storm, 1989; Gu et al., 2005; Honigsperger et al., 2015; Lombardo and Harrington, 2016; Dai et al., 2018). Presynaptically, KCNQ activation inhibits transmitter release (Wei and Ramirez, 2019). KCNQ activation during inspiratory bursts creates a shunt and progressively reduces membrane responsiveness to synaptic inputs (Brown, 1983; Brown and Passmore, 2009). Therefore, KCNQ at both pre- and postsynaptic sites in preBötC neurons can contribute to burst termination.

KCNQ modulation affected burst duration more than frequency in rhythmic slices. XE991 and linopirdine attenuate KCNQ and increase burst duration 2- to 3.5-fold. KCNQ potentiation *via* retigabine decreases burst duration ~30%. Regarding frequency, inhibition of KCNQ has minimal effects on frequency (5–20% decrease). Wei and Ramirez similarly found no effect of XE991 (≥50 μM) or Chromanol 293B (another KCNQ blocker) on inspiratory frequency in mouse slices (Wei and Ramirez, 2019). Potentiation of KCNQ with retigabine (or ICA 69673) also decreased frequency and blocked rhythm (Wei and Ramirez, 2019). In summary, inhibition and potentiation of KCNQ increase and decrease burst duration, respectively. In contrast, frequency modulation was unidirectional – inhibition of KCNQ did not affect frequency, but KCNQ potentiation decreased frequency.

What might account for these differential effects of KCNQ modulation on frequency and burst duration? We propose that frequency effects are primarily related to the influence of KCNQ on resting membrane potential, while the voltage- and time-dependent activation properties of KCNQ account for its effects on burst duration, consistent with Wei and Ramirez (2019). By contrast, modifications in [K^+^]_e_ potently modulate frequency yet exert a smaller effect on burst duration. Thus, this study focuses on KCNQ modulation of burst duration.

The postsynaptic actions of KCNQ can cause spike frequency adaptation, directly hyperpolarize the membrane potential, and shunt the membrane to synaptic drive, all of which can contribute to burst termination.

KCNQ activation during the inspiratory burst (hundreds of ms *in vitro*) progressively increases outward current (Brown, 1983; Brown and Passmore, 2009) that would counteract the ongoing excitation associated with inspiratory synaptic drive and activity of persistent currents such as persistent Na^+^ current (I_NaP_) and calcium-activated non-specific mixed cation current (I_CAN_; Pace et al., 2007; Del Negro et al., 2018; Picardo et al., 2019). Inspiratory burst termination requires a reversal of the balance of inward and outward currents toward outward. KCNQ has the biophysical properties to transiently shift this balance and thus extinguish ongoing burst-like activity, as documented in other systems. In superior cervical ganglion sympathetic neurons, the duration of action potential bursts is determined by the balance between I_NaP_ and KCNQ currents (Zaika et al., 2006). CA1 hippocampal neurons normally respond to short depolarizing excitatory current pulses with single action potentials, but those single spikes transform into bursts of action potentials in the presence of KCNQ inhibitor linopirdine (Yue and Yaari, 2004; Peters et al., 2005).

Sympathetic neurons discharge tonically or exhibit bursts of action potentials. Tonic neurons lack KCNQ, whereas bursting neurons express a large KCNQ current. Moreover, neurons that discharge in bursts switch to tonic firing after KCNQ is blocked (Wang and McKinnon, 1995; Wang et al., 1998). Those data indicate that KCNQ biophysics provide the activity-dependent outward current needed to transiently suppress spiking activity, which is obligatory for oscillatory bursting in single neurons, or as in the preBötC, a rhythmically active network.

It seems incongruent that the XE991-sensitive current measured 30–60 pA; i.e., this seems to be a relatively small current compared to the 2- to 3-fold increase in inspiratory burst duration evoked by KCNQ inhibition. Voltage clamp likely underestimates the true magnitude of the KCNQ current. First, there are voltage-clamp errors associated with uncompensated series resistance on the order of 12–16 MΩ. The maximum outward current evoked by voltage steps from −60 to −20 mV measured ~600 pA in one neuron and between 275 and 450 pA in the remaining four. By Ohm’s law, a 600-pA outward current across 12–16 MΩ of series resistance will result in a 7- to 12-mV error. Thus, a step from −60 to −20 mV would have clamped the membrane potential to between −30 and −23 mV, thus underestimating the magnitude of the XE991-sensitive (KCNQ) current.

An incomplete block of KCNQ by XE991 may also have contributed to an underestimation of KCNQ current magnitude. Prolonged (>30 min) incubation periods are required for XE991 to block KCNQ channels (Martire et al., 2004; Yue and Yaari, 2004; Gu et al., 2005; Peters et al., 2005; Yeung and Greenwood, 2005; Wladyka and Kunze, 2006; Leao et al., 2009), which is consistent with our experiments where >30 min was required for steady-state effects of XE991. Nevertheless, 30–60 min may have been insufficient to achieve a maximum block. In addition, KCNQ blockers were used at concentrations near their respective IC_50_ values (see below), which minimizes off-target actions but simultaneously ensures that the block would be incomplete.

It is also possible that the effects of XE991 on burst duration, recorded here from the XII nerve, are not mediated entirely through preBötC neurons but *via* other modulatory neurons or axons within the slice that project to preBötC, or through actions downstream from the preBötC at XII premotor neurons or motoneurons. Certainly, XII motoneurons express KCNQ channels, the blockade of which increases the number of spikes generated by a given depolarization (Ghezzi et al., 2017). Thus, for the experiments in which drugs were applied to the bath (Figures 3–5), we cannot exclude that KCNQ currents outside the preBötC contributed to the effects of KCNQ modulators on burst duration. However, the observation that the effects of XE991 on burst duration, recorded from the XII nerve, were similar whether applied to the bath or injected unilaterally into the preBötC (Figure 6) indicates that the majority of XE991 effects were mediated *via* neurons in the preBötC.

Finally, presynaptic KCNQ channel activation and the subsequent inhibition of transmitter release could contribute to the effects of KCNQ on burst duration but would not be measured by our voltage-clamp protocol in synaptically isolated preBötC neurons.

### Technical Limitations

Both XE991 and linopirdine have off-target actions. We used XE991 at a maximum concentration of 1 μM for bath application and 10–100 μM for local injection, which produces an effective concentration of ~1–10 μM (Liu et al., 1990). The IC_50_ for XE991 is ~1 μM (Wang et al., 1998). XE991 inhibits Kv2.1 in visceral sensory neurons. PreBötC neurons express Kv2.1 (Hayes et al., 2017) but at 10 μM XE991 blocks Kv2.1 by only ~7% (Wladyka and Kunze, 2006). XE991 also inhibits Kv4.3 transient K^+^ current in visceral sensory neurons (Wladyka and Kunze, 2006) and eag1 (ether-à-go-go) K^+^ channels in sympathetic cervical ganglion neurons (Wang et al., 1998). However, the IC_50_ for XE991 inhibition of these channels measure 43 ± 7 and 49 ± 6 μM, respectively (Wang et al., 1998). Thus, it is unlikely that XE991 effects on Kv2.1, Kv4.3, or eag1 influence respiratory rhythm and pattern *in vitro*.

Linopirdine was applied at 3 μM, and its IC_50_ is 3–7 μM (Costa and Brown, 1997; Lamas et al., 1997; Schnee and Brown, 1998; Wang et al., 1998). Linopirdine can inhibit large conductance Ca^2+^-activated K^+^ current (BK) in hippocampal neurons with an IC_50_ of ~16 μM (Lamas et al., 1997). However, off-target effects of linopirdine on BK current are unlikely in our context because more potent BK blockers show modest or inconsistent effects on inspiratory rhythm and pattern (reviewed in Rajani et al., 2016). Linopirdine also partially blocks leak, transient outward, and delayed rectifier K^+^ currents and the slow component of the AHP but at 100 μM, which exceeds our dosage by orders of magnitude (Schnee and Brown, 1998). Thus, it is unlikely that the actions of linopirdine were mediated by anything other than KCNQ inhibition.

### Physiological Significance

This study adds the KCNQ current to the list of candidate mechanisms that contribute to inspiratory burst termination in rodents. Of the four neuronal KCNQ isoforms (KCNQ2–5), isoforms 2–4 are the most likely to be involved in the preBötC because XE991 effectively blocks these isoforms with an IC_50_ < 10 μM while its IC_50_ at homomeric KCNQ5 channels is ~50 μM (Wang et al., 1998; Lerche et al., 2000; Schroeder et al., 2000; Schroder et al., 2001; Rajani et al., 2016). Data were generated using rhythmic medullary slices from newborn rat. Thus, the role of KCNQ in the intact, mature network remains to be explored. The role of KCNQ in burst termination relative to other mechanisms, including synaptic depression (Kottick and Del Negro, 2015) and Na^+^-dependent outward currents (Del Negro et al., 2009; Krey et al., 2010), also remains to be measured.

An additional point to consider is the potential role of KCNQ in the termination of burstlets, the putative rhythmogenic substrate, as opposed to bursts, which are obligatory for patterned motor output. Inspiratory rhythm generation is an emergent property of recurrently connected preBötC neurons that produce rhythmic “burstlets,” which are a subthreshold event from the standpoint of motor output. If network excitability is sufficiently high, then burstlets cross a threshold and trigger a full-amplitude burst that is transmitted out of the preBötC to premotoneurons, motoneurons, and inspiratory muscles (Kam et al., 2013; Del Negro et al., 2018; Ashhad and Feldman, 2020; Kallurkar et al., 2020). In the context of burst termination, synaptic depression, Na^+^/K^+^ ATPase, I_K-ATP_, and I_K-Na_ all depend on robust burst activity. But activity is much lower during a burstlet, and therefore, activity-dependent mechanisms may be less relevant in burstlet termination. KCNQ, in contrast, is voltage dependent and provides a compelling candidate that could contribute to burstlet termination as long as depolarization occurs.

## Data Availability Statement

The raw data supporting the conclusions of this article will be made available by the authors, for reanalysis or meta analyses.

## Ethics Statement

All experimental procedures were approved by the University of Alberta Faculty of Medicine Animal Welfare Committee and were performed in accordance with the Guidelines for the Care, Handling, and Treatment of Experimental Animals from the Canadian Council on Animal Care.

## Author Contributions

AR, AK, WM, and GF participated in the study design. AR and AK collected data. AR, AK, CDN, and GF wrote the manuscript. All authors were involved with manuscript revision, and read and approved the submitted version.

### Conflict of Interest

The authors declare that the research was conducted in the absence of any commercial or financial relationships that could be construed as a potential conflict of interest.

## References

[ref1] Ashhad S. Feldman J. L. (2020). Emergent elements of inspiratory rhythmogenesis: network synchronization and synchrony propagation. Neuron 106, 482.e4–497.e4. 10.1016/j.neuron.2020.02.005, PMID: 32130872 PMC11221628

[ref2] Baertsch N. A. Baertsch H. C. Ramirez J. M. (2018). The interdependence of excitation and inhibition for the control of dynamic breathing rhythms. Nat. Commun. 9:843. 10.1038/s41467-018-03223-x, PMID: 29483589 PMC5827754

[ref3] Bonham A. C. Coles S. K. McCrimmon D. R. (1993). Pulmonary stretch receptor afferents activate excitatory amino acid receptors in the nucleus tractus solitarii in rats. J. Physiol. 464, 725–745. 10.1113/jphysiol.1993.sp019660, PMID: 8229827 PMC1175411

[ref4] Brown D. A. (1983). Slow cholinergic excitation - a mechanism for increasing neuronal excitability. Trends Neurosci. 6, 302–307. 10.1016/0166-2236(83)90144-3

[ref5] Brown D. A. Adams P. R. (1980). Muscarinic suppression of a novel voltage-sensitive K^+^ current in a vertebrate neurone. Nature 283, 673–676. 10.1038/283673a0, PMID: 6965523

[ref6] Brown D. A. Passmore G. M. (2009). Neural KCNQ (Kv7) channels. Br. J. Pharmacol. 156, 1185–1195. 10.1111/j.1476-5381.2009.00111.x, PMID: 19298256 PMC2697739

[ref7] Costa A. M. Brown B. S. (1997). Inhibition of M-current in cultured rat superior cervical ganglia by linopirdine: mechanism of action studies. Neuropharmacology 36, 1747–1753. 10.1016/S0028-3908(97)00155-X, PMID: 9517447

[ref8] Cregg J. M. Chu K. A. Dick T. E. Landmesser L. T. Silver J. (2017). Phasic inhibition as a mechanism for generation of rapid respiratory rhythms. Proc. Natl. Acad. Sci. U. S. A. 114, 12815–12820. 10.1073/pnas.1711536114, PMID: 29133427 PMC5715763

[ref9] Crowder E. A. Saha M. S. Pace R. W. Zhang H. Prestwich G. D. Del Negro C. A. (2007). Phosphatidylinositol 4,5-bisphosphate regulates inspiratory burst activity in the neonatal mouse preBotzinger complex. J. Physiol. 582, 1047–1058. 10.1113/jphysiol.2007.134577, PMID: 17599963 PMC2075248

[ref10] Cui Y. Kam K. Sherman D. Janczewski W. A. Zheng Y. Feldman J. L. (2016). Defining preBotzinger complex rhythm- and pattern-generating neural microcircuits in vivo. Neuron 91, 602–614. 10.1016/j.neuron.2016.07.003, PMID: 27497222 PMC4978183

[ref11] Dai Y. Cheng Y. Fedirchuk B. Jordan L. M. Chu J. (2018). Motoneuron output regulated by ionic channels: a modeling study of motoneuron frequency-current relationships during fictive locomotion. J. Neurophysiol. 120, 1840–1858. 10.1152/jn.00068.2018, PMID: 30044677

[ref12] Del Negro C. A. Funk G. D. Feldman J. L. (2018). Breathing matters. Nat. Rev. Neurosci. 19, 351–367. 10.1038/s41583-018-0003-6, PMID: 29740175 PMC6636643

[ref13] Del Negro C. A. Johnson S. M. Butera R. J. Smith J. C. (2001). Models of respiratory rhythm generation in the pre-Botzinger complex. III. Experimental tests of model predictions. J. Neurophysiol. 86, 59–74. 10.1152/jn.2001.86.1.59, PMID: 11431488

[ref14] Del Negro C. A. Kam K. Hayes J. A. Feldman J. L. (2009). Asymmetric control of inspiratory and expiratory phases by excitability in the respiratory network of neonatal mice in vitro. J. Physiol. 587, 1217–1231. 10.1113/jphysiol.2008.164079, PMID: 19171658 PMC2674993

[ref15] Delmas P. Brown D. A. (2005). Pathways modulating neural KCNQ/M (Kv7) potassium channels. Nat. Rev. Neurosci. 6, 850–862. 10.1038/nrn1785, PMID: 16261179

[ref16] Feldman J. L. Del Negro C. A. (2006). Looking for inspiration: new perspectives on respiratory rhythm. Nat. Rev. Neurosci. 7, 232–242. 10.1038/nrn1871, PMID: 16495944 PMC2819067

[ref17] Feldman J. L. Smith J. C. (1989). Cellular mechanisms underlying modulation of breathing pattern in mammals. Ann. N. Y. Acad. Sci. 563, 114–130. 10.1111/j.1749-6632.1989.tb42194.x, PMID: 2476055

[ref18] Feldman J. L. Windhorst U. Anders K. Richter D. W. (1992). Synaptic interaction between medullary respiratory neurones during apneusis induced by NMDA-receptor blockade in cat. J. Physiol. 450, 303–323. 10.1113/jphysiol.1992.sp019128, PMID: 1432710 PMC1176123

[ref19] Foutz A. S. Champagnat J. Denavit-Saubie M. (1988). N-methyl-D-aspartate (NMDA) receptors control respiratory off-switch in cat. Neurosci. Lett. 87, 221–226. 10.1016/0304-3940(88)90452-1, PMID: 2837690

[ref20] Funk G. D. Greer J. J. (2013). The rhythmic, transverse medullary slice preparation in respiratory neurobiology: contributions and caveats. Respir. Physiol. Neurobiol. 186, 236–253. 10.1016/j.resp.2013.01.011, PMID: 23357617

[ref21] Funk G. D. Smith J. C. Feldman J. L. (1995). Modulation of neural network activity in vitro by cyclothiazide, a drug that blocks desensitization of AMPA receptors. J. Neurosci. 15, 4046–4056. 10.1523/JNEUROSCI.15-05-04046.1995, PMID: 7751964 PMC6578237

[ref22] Ghezzi F. Corsini S. Nistri A. (2017). Electrophysiological characterization of the M-current in rat hypoglossal motoneurons. Neuroscience 340, 62–75. 10.1016/j.neuroscience.2016.10.048, PMID: 27984184

[ref23] Greene D. L. Kang S. Hoshi N. (2017). XE991 and linopirdine are state-dependent inhibitors for Kv7/KCNQ channels that favor activated single subunits. J. Pharmacol. Exp. Ther. 362, 177–185. 10.1124/jpet.117.241679, PMID: 28483800 PMC5478917

[ref24] Grillner S. (2006). Biological pattern generation: the cellular and computational logic of networks in motion. Neuron 52, 751–766. 10.1016/j.neuron.2006.11.008, PMID: 17145498

[ref25] Grinton B. E. Heron S. E. Pelekanos J. T. Zuberi S. M. Kivity S. Afawi Z.. (2015). Familial neonatal seizures in 36 families: clinical and genetic features correlate with outcome. Epilepsia 56, 1071–1080. 10.1111/epi.13020, PMID: 25982755

[ref26] Gu N. Vervaeke K. Hu H. Storm J. F. (2005). Kv7/KCNQ/M and HCN/h, but not KCa2/SK channels, contribute to the somatic medium after-hyperpolarization and excitability control in CA1 hippocampal pyramidal cells. J. Physiol. 566, 689–715. 10.1113/jphysiol.2005.086835, PMID: 15890705 PMC1464792

[ref27] Guerrier C. Hayes J. A. Fortin G. Holcman D. (2015). Robust network oscillations during mammalian respiratory rhythm generation driven by synaptic dynamics. Proc. Natl. Acad. Sci. U. S. A. 112, 9728–9733. 10.1073/pnas.1421997112, PMID: 26195782 PMC4534249

[ref28] Gunthorpe M. J. Large C. H. Sankar R. (2012). The mechanism of action of retigabine (ezogabine), a first-in-class K^+^ channel opener for the treatment of epilepsy. Epilepsia 53, 412–424. 10.1111/j.1528-1167.2011.03365.x, PMID: 22220513

[ref29] Harris M. B. Milsom W. K. (2003). Apneusis follows disruption of NMDA-type glutamate receptors in vagotomized ground squirrels. Respir. Physiol. Neurobiol. 134, 191–207. 10.1016/S1569-9048(02)00223-9, PMID: 12660099

[ref30] Hayashi F. Lipski J. (1992). The role of inhibitory amino acids in control of respiratory motor output in an arterially perfused rat. Respir. Physiol. 89, 47–63. 10.1016/0034-5687(92)90070-D, PMID: 1325666

[ref31] Hayes J. A. Kottick A. Picardo M. C. D. Halleran A. D. Smith R. D. Smith G. D.. (2017). Transcriptome of neonatal preBotzinger complex neurones in Dbx1 reporter mice. Sci. Rep. 7:8669. 10.1038/s41598-017-09418-4, PMID: 28819234 PMC5561182

[ref32] Honigsperger C. Marosi M. Murphy R. Storm J. F. (2015). Dorsoventral differences in Kv7/M-current and its impact on resonance, temporal summation and excitability in rat hippocampal pyramidal cells. J. Physiol. 593, 1551–1580. 10.1113/jphysiol.2014.280826, PMID: 25656084 PMC4386960

[ref33] Ireland M. F. Funk G. D. Bellingham M. C. (2012). Muscarinic acetylcholine receptors enhance neonatal mouse hypoglossal motoneuron excitability in vitro. J. Appl. Physiol. 113, 1024–1039. 10.1152/japplphysiol.00699.2011, PMID: 22858620

[ref34] Janczewski W. A. Tashima A. Hsu P. Cui Y. Feldman J. L. (2013). Role of inhibition in respiratory pattern generation. J. Neurosci. 33, 5454–5465. 10.1523/JNEUROSCI.1595-12.2013, PMID: 23536061 PMC3724454

[ref35] Kallurkar P. S. Grover C. Picardo M. C. D. Del Negro C. A. (2020). Evaluating the Burstlet theory of inspiratory rhythm and pattern generation. eNeuro 7:ENEURO.0314-19.2019. 10.1523/ENEURO.0314-19.2019, PMID: 31888961 PMC6964920

[ref36] Kam K. Worrell J. W. Janczewski W. A. Cui Y. Feldman J. L. (2013). Distinct inspiratory rhythm and pattern generating mechanisms in the preBotzinger complex. J. Neurosci. 33, 9235–9245. 10.1523/JNEUROSCI.4143-12.2013, PMID: 23719793 PMC3737080

[ref37] Kato M. Yamagata T. Kubota M. Arai H. Yamashita S. Nakagawa T.. (2013). Clinical spectrum of early onset epileptic encephalopathies caused byKCNQ2mutation. Epilepsia 54, 1282–1287. 10.1111/epi.12200, PMID: 23621294

[ref38] Koizumi H. Wilson C. G. Wong S. Yamanishi T. Koshiya N. Smith J. C. (2008). Functional imaging, spatial reconstruction, and biophysical analysis of a respiratory motor circuit isolated in vitro. J. Neurosci. 28, 2353–2365. 10.1523/JNEUROSCI.3553-07.2008, PMID: 18322082 PMC6671179

[ref39] Kottick A. Del Negro C. A. (2015). Synaptic depression influences inspiratory-expiratory phase transition in Dbx1 interneurons of the preBotzinger complex in neonatal mice. J. Neurosci. 35, 11606–11611. 10.1523/JNEUROSCI.0351-15.2015, PMID: 26290237 PMC4540798

[ref40] Krey R. A. Goodreau A. M. Arnold T. B. Del Negro C. A. (2010). Outward currents contributing to inspiratory burst termination in preBotzinger complex neurons of neonatal mice studied in vitro. Front. Neural Circuits 4:124. 10.3389/fncir.2010.00124, PMID: 21151816 PMC2999835

[ref41] Lamas J. A. Selyanko A. A. Brown D. A. (1997). Effects of a cognition-enhancer, linopirdine (DuP 996), on M-type potassium currents (IK(M)) and some other voltage- and ligand-gated membrane currents in rat sympathetic neurons. Eur. J. Neurosci. 9, 605–616. 10.1111/j.1460-9568.1997.tb01637.x, PMID: 9104602

[ref42] Leao R. N. Tan H. M. Fisahn A. (2009). Kv7/KCNQ channels control action potential phasing of pyramidal neurons during hippocampal gamma oscillations in vitro. J. Neurosci. 29, 13353–13364. 10.1523/JNEUROSCI.1463-09.2009, PMID: 19846723 PMC6665214

[ref43] Lerche C. Scherer C. R. Seebohm G. Derst C. Wei A. D. Busch A. E.. (2000). Molecular cloning and functional expression of KCNQ5, a potassium channel subunit that may contribute to neuronal M-current diversity. J. Biol. Chem. 275, 22395–22400. 10.1074/jbc.M002378200, PMID: 10787416

[ref44] Liu G. Feldman J. L. Smith J. C. (1990). Excitatory amino acid-mediated transmission of inspiratory drive to phrenic motoneurons. J. Neurophysiol. 64, 423–436. 10.1152/jn.1990.64.2.423, PMID: 1976765

[ref45] Lombardo J. Harrington M. A. (2016). Nonreciprocal mechanisms in up- and downregulation of spinal motoneuron excitability by modulators of KCNQ/Kv7 channels. J. Neurophysiol. 116, 2114–2124. 10.1152/jn.00446.2016, PMID: 27512022 PMC5102305

[ref46] Lorier A. R. Huxtable A. G. Robinson D. M. Lipski J. Housley G. D. Funk G. D. (2007). P2Y1 receptor modulation of the pre-Botzinger complex inspiratory rhythm generating network in vitro. J. Neurosci. 27, 993–1005. 10.1523/JNEUROSCI.3948-06.2007, PMID: 17267553 PMC6673186

[ref47] Lumsden T. (1923). Observations on the respiratory centres in the cat. J. Physiol. 57, 153–160. 10.1113/jphysiol.1923.sp002052, PMID: 16993609 PMC1405470

[ref48] Marrion N. V. (1997). Control of M-current. Annu. Rev. Physiol. 59, 483–504. 10.1146/annurev.physiol.59.1.483, PMID: 9074774

[ref49] Martire M. Castaldo P. D’Amico M. Preziosi P. Annunziato L. Taglialatela M. (2004). M channels containing KCNQ2 subunits modulate norepinephrine, aspartate, and GABA release from hippocampal nerve terminals. J. Neurosci. 24, 592–597. 10.1523/JNEUROSCI.3143-03.2004, PMID: 14736843 PMC6729253

[ref50] Onimaru H. Ballanyi K. Homma I. (2003). Contribution of Ca^2+^-dependent conductances to membrane potential fluctuations of medullary respiratory neurons of newborn rats in vitro. J. Physiol. 552, 727–741. 10.1113/jphysiol.2003.049312, PMID: 12937288 PMC2343467

[ref51] Pace R. W. Mackay D. D. Feldman J. L. Del Negro C. A. (2007). Inspiratory bursts in the preBotzinger complex depend on a calcium-activated non-specific cation current linked to glutamate receptors in neonatal mice. J. Physiol. 582, 113–125. 10.1113/jphysiol.2007.133660, PMID: 17446214 PMC2075310

[ref52] Peters H. C. Hu H. Pongs O. Storm J. F. Isbrandt D. (2005). Conditional transgenic suppression of M channels in mouse brain reveals functions in neuronal excitability, resonance and behavior. Nat. Neurosci. 8, 51–60. 10.1038/nn1375, PMID: 15608631

[ref53] Picardo M. C. D. Sugimura Y. K. Dorst K. E. Kallurkar P. S. Akins V. T. Ma X.. (2019). Trpm4 ion channels in pre-Botzinger complex interneurons are essential for breathing motor pattern but not rhythm. PLoS Biol. 17:e2006094. 10.1371/journal.pbio.2006094, PMID: 30789900 PMC6400419

[ref54] Ptak K. Yamanishi T. Aungst J. Milescu L. S. Zhang R. Richerson G. B.. (2009). Raphe neurons stimulate respiratory circuit activity by multiple mechanisms via endogenously released serotonin and substance P. J. Neurosci. 29, 3720–3737. 10.1523/JNEUROSCI.5271-08.2009, PMID: 19321769 PMC2940110

[ref55] Rajani V. Zhang Y. Jalubula V. Rancic V. SheikhBahaei S. Zwicker J. D.. (2018). Release of ATP by pre-Botzinger complex astrocytes contributes to the hypoxic ventilatory response via a Ca^(2+)^ -dependent P2Y1 receptor mechanism. J. Physiol. 596, 3245–3269. 10.1113/JP274727, PMID: 28678385 PMC6068109

[ref56] Rajani V. Zhang Y. Revill A. L. Funk G. D. (2016). The role of P2Y1 receptor signaling in central respiratory control. Respir. Physiol. Neurobiol. 226, 3–10. 10.1016/j.resp.2015.10.003, PMID: 26476057

[ref57] Rekling J. C. Champagnat J. Denavit-Saubie M. (1996). Electroresponsive properties and membrane potential trajectories of three types of inspiratory neurons in the newborn mouse brain stem in vitro. J. Neurophysiol. 75, 795–810. 10.1152/jn.1996.75.2.795, PMID: 8714653

[ref58] Revill A. L. Vann N. C. Akins V. T. Kottick A. Gray P. A. Del Negro C. A.. (2015). Dbx1 precursor cells are a source of inspiratory XII premotoneurons. Elife 4:e12301. 10.7554/eLife.12301, PMID: 26687006 PMC4764567

[ref59] Ronen G. M. Rosales T. O. Connolly M. Anderson V. E. Leppert M. (1993). Seizure characteristics in chromosome 20 benign familial neonatal convulsions. Neurology 43, 1355–1360. 10.1212/WNL.43.7.1355, PMID: 8327138

[ref60] Ruangkittisakul A. Schwarzacher S. W. Secchia L. Poon B. Y. Ma Y. Funk G. D.. (2006). High sensitivity to neuromodulator-activated signaling pathways at physiological [K^+^] of confocally imaged respiratory center neurons in on-line-calibrated newborn rat brainstem slices. J. Neurosci. 26, 11870–11880. 10.1523/JNEUROSCI.3357-06.2006, PMID: 17108160 PMC6674865

[ref61] Rubin J. E. Hayes J. A. Mendenhall J. L. Del Negro C. A. (2009). Calcium-activated nonspecific cation current and synaptic depression promote network-dependent burst oscillations. Proc. Natl. Acad. Sci. U. S. A. 106, 2939–2944. 10.1073/pnas.0808776106, PMID: 19196976 PMC2636730

[ref62] Schnee M. E. Brown B. S. (1998). Selectivity of linopirdine (DuP 996), a neurotransmitter release enhancer, in blocking voltage-dependent and calcium-activated potassium currents in hippocampal neurons. J. Pharmacol. Exp. Ther. 286, 709–717. PMID: 9694925

[ref63] Schroder R. L. Jespersen T. Christophersen P. Strobaek D. Jensen B. S. Olesen S. P. (2001). KCNQ4 channel activation by BMS-204352 and retigabine. Neuropharmacology 40, 888–898. 10.1016/S0028-3908(01)00029-6, PMID: 11378159

[ref64] Schroeder B. C. Hechenberger M. Weinreich F. Kubisch C. Jentsch T. J. (2000). KCNQ5, a novel potassium channel broadly expressed in brain, mediates M-type currents. J. Biol. Chem. 275, 24089–24095. 10.1074/jbc.M003245200, PMID: 10816588

[ref65] Selyanko A. A. Stansfeld C. E. Brown D. A. (1992). Closure of potassium M-channels by muscarinic acetylcholine-receptor stimulants requires a diffusible messenger. Proc. Roy. Soc. B-Biol. Sci. 250, 119–125. 10.1098/rspb.1992.0139 1361985

[ref66] Sherman D. Worrell J. W. Cui Y. Feldman J. L. (2015). Optogenetic perturbation of preBotzinger complex inhibitory neurons modulates respiratory pattern. Nat. Neurosci. 18, 408–414. 10.1038/nn.3938, PMID: 25643296 PMC4340826

[ref67] Smith J. C. Ellenberger H. H. Ballanyi K. Richter D. W. Feldman J. L. (1991). Pre-Botzinger complex: a brainstem region that may generate respiratory rhythm in mammals. Science 254, 726–729. 10.1126/science.1683005, PMID: 1683005 PMC3209964

[ref68] Smith J. C. Greer J. J. Liu G. S. Feldman J. L. (1990). Neural mechanisms generating respiratory pattern in mammalian brain stem-spinal cord in vitro. I. Spatiotemporal patterns of motor and medullary neuron activity. J. Neurophysiol. 64, 1149–1169. 10.1152/jn.1990.64.4.1149, PMID: 2258739

[ref69] Stella G. (1938). On the mechanism of production, and the physiological significance of “apneusis”. J. Physiol. 93, 10–23. 10.1113/jphysiol.1938.sp003621, PMID: 16994990 PMC1393517

[ref70] Storm J. F. (1989). An after-hyperpolarization of medium duration in rat hippocampal pyramidal. Cells 409, 171–190. 10.1113/jphysiol.1989.sp017491 PMC1190438 2585290

[ref71] Thoby-Brisson M. Telgkamp P. Ramirez J. M. (2000). The role of the hyperpolarization-activated current in modulating rhythmic activity in the isolated respiratory network of mice. J. Neurosci. 20, 2994–3005. 10.1523/JNEUROSCI.20-08-02994.2000, PMID: 10751452 PMC6772196

[ref72] von Euler C. Marttila I. Remmers J. E. Trippenbach T. (1976). Effects of lesions in the parabrachial nucleus on the mechanisms for central and reflex termination of inspiration in the cat. Acta Physiol. Scand. 96, 324–337. 10.1111/j.1748-1716.1976.tb10203.x, PMID: 1274615

[ref73] Wang H. S. McKinnon D. (1995). Potassium currents in rat prevertebral and paravertebral sympathetic neurones: control of firing properties. J. Physiol. 485, 319–335. 10.1113/jphysiol.1995.sp020732, PMID: 7666361 PMC1157995

[ref74] Wang H. S. Pan Z. Shi W. Brown B. S. Wymore R. S. Cohen I. S.. (1998). KCNQ2 and KCNQ3 potassium channel subunits: molecular correlates of the M-channel. Science 282, 1890–1893. 10.1126/science.282.5395.1890, PMID: 9836639

[ref75] Watanabe H. Nagata E. Kosakai A. Nakamura M. Yokoyama M. Tanaka K.. (2001). Disruption of the epilepsy KCNQ2 gene results in neural hyperexcitability. J. Neurochem. 75, 28–33. 10.1046/j.1471-4159.2000.0750028.x, PMID: 10854243

[ref76] Wei A. D. Ramirez J. M. (2019). Presynaptic mechanisms and KCNQ potassium channels modulate opioid depression of respiratory drive. Front. Physiol. 10:1407. 10.3389/fphys.2019.01407, PMID: 31824331 PMC6882777

[ref77] Wladyka C. L. Kunze D. L. (2006). KCNQ/M-currents contribute to the resting membrane potential in rat visceral sensory neurons. J. Physiol. 575, 175–189. 10.1113/jphysiol.2006.113308, PMID: 16777937 PMC1819429

[ref78] Yeung S. Y. Greenwood I. A. (2005). Electrophysiological and functional effects of the KCNQ channel blocker XE991 on murine portal vein smooth muscle cells. Br. J. Pharmacol. 146, 585–595. 10.1038/sj.bjp.0706342, PMID: 16056238 PMC1751185

[ref79] Yue C. Yaari Y. (2004). KCNQ/M channels control spike afterdepolarization and burst generation in hippocampal neurons. J. Neurosci. 24, 4614–4624. 10.1523/JNEUROSCI.0765-04.2004, PMID: 15140933 PMC6729392

[ref80] Zaika O. Lara L. S. Gamper N. Hilgemann D. W. Jaffe D. B. Shapiro M. S. (2006). Angiotensin II regulates neuronal excitability via phosphatidylinositol 4,5-bisphosphate-dependent modulation of Kv7 (M-type) K^+^ channels. J. Physiol. 575, 49–67. 10.1113/jphysiol.2006.114074, PMID: 16777936 PMC1819424

[ref81] Zwicker J. D. Rajani V. Hahn L. B. Funk G. D. (2011). Purinergic modulation of preBotzinger complex inspiratory rhythm in rodents: the interaction between ATP and adenosine. J. Physiol. 589, 4583–4600. 10.1113/jphysiol.2011.210930, PMID: 21788352 PMC3208226

